# "Puppy Escape" ‐ A Novel Narrative Recall Task Leveraging Multimodal Analysis and Weighted Scoring

**DOI:** 10.1002/alz70857_105518

**Published:** 2025-12-25

**Authors:** Michael J Kleiman, Mirza Mohmmed Baig, James E. Galvin

**Affiliations:** ^1^ University of Miami Miller School of Medicine, Boca Raton, FL, USA

## Abstract

**Background:**

Narrative recall tasks effectively differentiate between individuals with and without cognitive impairment, particularly when comparing recall after a 30 minute delay. However, such substantial delays are prohibitive in many clinical contexts, especially in small or remote clinics. While immediate narrative recall outperforms delayed recall for identifying subtle changes in preclinical and very mild impairment, most existing narratives include simple details designed for longer delays. To address this limitation, we developed the “Puppy Escape” story, a novel narrative with assessment criteria focused on a shorter 15‐minute delay, incorporating both verbatim and paraphrased responses in the weighted scoring system.

**Method:**

93 participants were included: 60 cognitively normal (CN), 8 preclinical AD (pAD), and 25 mild cognitive impairment (MCI), with no significant differences in age, sex, or years of education. The narrative was presented as a 45‐second audio narration with visual text presentation to encourage multimodal encoding. Analyses included ANOVAs with Tukey post hoc tests, pairwise logistic regressions, and machine learning models incorporating lexical and audiometric data alongside a score generated from both general and specific criteria with variable weighting.

**Result:**

ANOVA revealed significant differences in immediate recall between diagnostic groups (F(2,90)=12.37, *p* < .001), particularly between CN and MCI (*p* < .001) and CN and pAD (*p* = .026). Delayed recall (15 minutes) showed significance (F(2,90)=12.24, *p* < .001) only between CN and MCI (*p* < .001). Logistic regressions predicting MCI and pAD from CN were significant for both immediate (*r* ^2^=‐.13, *p* < .001; *r* ^2^=‐0.10, *p* = .023) and delayed recall (*r* ^2^ = .10, *p* < .001; *r* ^2^=‐.063, *p* = .043). The best‐performing machine learning model, incorporating weighted scores with acoustic and lexical features, achieved 72.7% sensitivity, 94.0% specificity, F1 of 0.76, and AUC of 0.870.

**Conclusion:**

The “Puppy Escape” narrative, freely available for research purposes, demonstrates effectiveness with only a 15‐minute delay. Its enhanced performance is attributed to its longer length (124 words versus Craft Story's 57 words) and multimodal presentation, enabling detection of subtle executive function degradation in mild cognitive impairment. Ongoing work includes additional data collection and feature engineering to maintain high discriminative ability in machine learning models.

